# Unveiling the Role of Donor Impurity Position on the Electronic Properties in Strained Type I and Type II Core/Shell Quantum Dots under Magnetic Field

**DOI:** 10.3390/ma16196535

**Published:** 2023-10-02

**Authors:** Laura M. Pérez, Noreddine Aghoutane, David Laroze, Pablo Díaz, Mohamed El-Yadri, El Mustapha Feddi

**Affiliations:** 1 Departamento de Física, FACI, Universidad de Tarapacá, Casilla 7D, Arica 1000000, Chile; 2 Instituto de Alta Investigación, Universidad de Tarapacá, Casilla 7D, Arica 1000000, Chile; noreddine.aghoutane@gmail.com (N.A.); dlarozen@uta.cl (D.L.); 3 Departamento de Ciencias Físicas, Universidad de La Frontera, Casilla 54-D, Temuco 4780000, Chile; pablo.diaz@ufrontera.cl; 4 Group of Optoelectronic of Semiconductors and Nanomaterials, ENSAM, Mohammed V University, Rabat 10100, Moroccoe.feddi@um5r.ac.ma (E.M.F.); 5 Institute of Applied Physics, Mohammed VI Polytechnic University, Ben Guerir 43150, Morocco

**Keywords:** type I and type II core/shell, impurity, diamagnetic susceptibility, binding energy, magnetic field

## Abstract

In this theoretical investigation, we delve into the significant effects of donor impurity position within core/shell quantum dot structures: type I (CdTe/ZnS) and type II (CdTe/CdS). The donor impurity’s precise location within both the core and the shell regions is explored to unveil its profound influence on the electronic properties of these nanostructures. Our study investigates the diamagnetic susceptibility and binding energy of the donor impurity while considering the presence of an external magnetic field. Moreover, the lattice mismatch-induced strain between the core and shell materials is carefully examined as it profoundly influences the electronic structure of the quantum dot system. Through detailed calculations, we analyze the strain effects on the conduction and valence bands, as well as the electron and hole energy spectrum within the core/shell quantum dots. The results highlight the significance of donor impurity position as a key factor in shaping the behaviors of impurity binding energy and diamagnetic susceptibility. Furthermore, our findings shed light on the potential for tuning the electronic properties of core/shell quantum dots through precise impurity positioning and strain engineering.

## 1. Introduction

In recent decades, nanometer-scale semiconductor systems, such as quantum dots (QDs), have garnered significant attention due to their unique electronic properties and similarities to the atom. Thus, quantifying the electronic states of confined charge carriers has opened the way to various practical applications [1,2,3,4,5,6]. Nowadays, with the advancement of manufacturing processes, new architectural structures called core/shell QD (CSQD) can be constructed from two materials with different bandgaps (one is the core, and the other is the shell). Indeed, due to lattice mismatch between the semiconductor materials, the strain field arising from the material growth to form core/shell QD can deeply influence the band structure. Consequently, this strain plays a significant role in the electronic and optical properties of these structures [7,8]. According to the band alignments, the core/shell structures are classified into two principal types: type I (particles are inside the core) [9], and type II (one of the particles is confined in the core while the other is in the shell) [10]. Based on the band gap engineering, the energy levels of electrons or holes confined in these nanostructures (CSQD) can be controlled via the sizes and shapes of the core or/and shell. In addition, the CSQD systems exhibit stronger photoluminescence and extremely high stability because the particles and quasi-particles (electrons, holes, impurities, excitons, excitonic complexes) within these structures are better isolated from the surface effects.

Among the different particles and quasi-particles in the QD structures, impurities take a special position, which plays a significant role in controlling various QD properties. Since several theoretical and experimental studies have focused on the impurities complexes within QD with different sizes and shapes, such as neutral and negative donor impurities [11], exciton trapped by an ionized donor ($D^{+},X$) [12], ionized double-donor complex ($D_{2}^{+}$) [13,14]. In addition, the effects of exteriors perturbations such as magnetic, electric, and intense laser fields, pressure, and temperature on the electric and optical properties of the particles trapped on QD have been widely investigated in the literature [15,16,17,18,19,20,21,22,23,24,25]. Niculescu et al. [26] have examined the impact of the dome-shaped QD’s impurity state dependence on the magnetic field; they found that the ground state energy’s diamagnetic shift increases monotonically with the applied field. Solaimani has investigated the diamagnetic susceptibility and binding energy of donor impurities in QD for various potentials and geometries [27]. Another study was performed by Saha et al. regarding the influence of pressure and temperature on an impurity’s diamagnetic susceptibility in QD under the aegis of noise [28]. The diamagnetic susceptibility of a magneto-donor in Inhomogeneous QD was also treated by Mmadi et al. [29]; their findings demonstrate that the magnetic field increases both diamagnetic susceptibility and binding energy.

Regarding the CSQD, numerous works have been devoted to these structures-either type I or type II, for *CdTe/ZnS*, *CdTe/CdS* among other materials, in recent years [30,31,32,33,34,35,36,37,38,39,40,41,42,43,44,45,46,47]. The strain field effect, resulting from the lattice mismatches, on CSQD has been performed in some works [48,49,50,51]. Concerning impurities confined in CSQD systems, Talbi et al. have studied the effect of LO-Phonons and dielectric polarization on impurity properties in GaN/InN spherical CSQD [52]; their results show that both parameters have relatively important contributions to the impurity binding energy. El-Yadri et al. have carried out the influences of temperature and pressure on single dopant states in hollow cylindrical CSQD [53]; their results demonstrate that temperature and pressure have opposite effects on impurity’s photoionization cross-section and binding energy. Merwyn et al. have examined the diamagnetic susceptibility of low-lying states of a donor impurity in a GaAs/Al${}_{1-x}$Ga${}_{x}$As CSQD [54]. Hayrapetyan et al. have investigated the effect of pressure on the diamagnetic susceptibility of impurity in core/shell/shell QD with Kratzer confining potential; they demonstrated that the diamagnetic susceptibility grows with the increase of the pressure [55].

However, looking closely into these research studies, we note that they overlook the impact of the critical radius and strain field resulting from the lattice mismatches, which have a crucial effects on the electronic properties of the particles. In connection with this topic, our work is interested in the study of impurity’s diamagnetic susceptibility $\chi_{dia}$ and binding energy $E_{b}$ in the presence of a magnetic field for two specific core/shell materials, CdTe/ZnS and CdTe/CdS. Our study takes into account the effects of impurity position, and the dimensions of the core and shell regions. By investigating these factors, we aim to provide novel insights into the electronic properties of strained core/shell quantum dots and their response to magnetic fields. By employing numerical calculations within the effective-mass approximation, we explore the effects of impurity position, magnetic field, and core/shell dimensions on the binding energy and diamagnetic susceptibility. The remainder of this paper is organized as follows: Section 2 provides detailed theoretical background on the electronic structure of core/shell quantum dots and the calculation methodology. Section 3 presents the results and discussion, focusing on the behavior of electron and hole energies, binding energy, and diamagnetic susceptibility as a function of various parameters. Finally, Section 4 summarizes the main findings of our study and discusses their implications. Through this work, we aim to advance the understanding of impurity behavior in strained core/shell quantum dots and inspire further investigations in this exciting field.

## 2. Background Theory

### 2.1. One Particle in Core/Shell Structures

**Figure 1:**
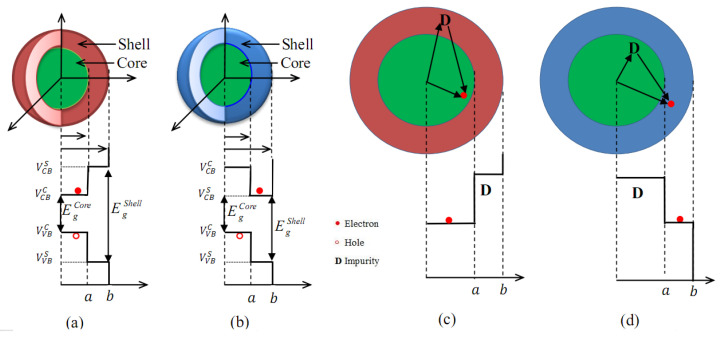
Pictorial view of type I (**a**) and type II (**b**) core/shell nanostructure with their related band offsets. (**c**,**d**) show the schematic representation of type I and type II, respectively, in the presence of the impurity. Only the cases of bound states of charge carriers ($E_{i}<V_{i}$) were represented. The full picture of all possible positions of particles is given in ref. [56].

We begin our study by examining the case of one particle (electron (*e*) and hole (*h*)) confined in two different spherical core/shell quantum dots (type I and type II), composed by a core material (with a dielectric constant $\varepsilon^{c}$ and radius *a*) over-coated by another shell material (with a dielectric constant $\varepsilon^{s}$ and radius b). For one particle, the Hamiltonian can be written as:

(1) $$H_{i}=-\frac{\hbar^{2}}{2m_{i}^{*}}\Delta_{i}+V_{i},\;\;\left(i=e,h\right)$$

where $m_{i}^{*}$ is the effective mass of the particle *i* (*i* = *e*, *h*) defined as follows:

(2) $$m_{i}^{*}\left(r_{i}\right)=\left\{\begin{array}{c} m_{ic}^{*}\;\mathrm{for}\;0<r_{i}<a,\\ m_{is}^{*}\;\mathrm{for}\;a<r_{i}<b, \end{array}\right.$$

and $V_{i}$ denotes the position-dependent confinement potentials of particle *i* (*i* = *e*, *h*). The corresponding confinement potential for type I CSQD (Figure 1a) takes the following forms:

(3) $$V_{i}=\left\{\begin{array}{c} 0,\;\mathrm{for}\;0<r_{e}<a\\ V_{i},\;\mathrm{for}\;a<r_{e}<b\\ \infty ,\;\;\mathrm{otherwise}. \end{array}\right.,\;i=e,h$$

For the case of the type II CSQD (Figure 1b), the corresponding confinement potentials for electrons *e* and holes *h* have a different form and are given by:

(4) $$V_{e}=\left\{\begin{array}{c} V_{e},\;\mathrm{for}\;0<r_{e}<a\\ 0,\;\mathrm{for}\;a<r_{e}<b\\ \infty ,\;\;\mathrm{otherwise}; \end{array}\right.$$

(5) $$V_{h}=\left\{\begin{array}{c} 0,\;\mathrm{for}\;0<r_{h}<a\\ V_{h},\;\mathrm{for}\;a<r_{h}<b\\ \infty ,\;\;\mathrm{otherwise}. \end{array}\right.$$

First, we should point out that the possibility of particles existing in both the core and the shell complicates the solution to the problem of charge carriers trapped in a CSQD structure. This complication arises from the fact that the location of the particles is highly dependent on the band offsets ($V_{i}$). In a previous work, this issue has been addressed in detail [56].

Continuing, the solution of the Schrödinger equation for a particle, using spherical coordinates (r,θ,φ) with separation of variables, takes the form given by:

(6) $$\psi_{i}\left(r_{i}\right)=R_{n,l}^{i}\left(r_{i}\right)Y_{l,m}^{i}\left(\theta ,\;\varphi \right),\;\;i=e,\;h$$

where $R_{n,l}^{i}\left(r_{i}\right)$ is the radial part and $Y_{l,m}^{i}\left(\theta ,\varphi \right)$ is the spherical harmonic function which equals to $1/\sqrt{4\pi }$ for the fundamental state (n=1, l=m=0).

As we have already mentioned, solving the Schrödinger equation in the two possible positions of the particles is necessary to find out their wave functions in the core/shell structure. The explicit expressions of the radial part of the ground state wave function are as follows for the type I CSQD structure:

(7) $$R_{n,l}^{i}\left(r_{i}\right)=\left\{\begin{array}{c} A_{i}^{c}\frac{\sin(k_{i}^{c}r_{i})}{r_{i}},\;0<r_{e}<a,\;i=e,h\\ A_{i}^{s}\frac{\sinh(k_{i}^{s1}\left(r_{i}-b\right))}{r_{i}}\;,a<r_{e}<b,\;E_{i}<V_{i}\\ A_{i}^{s}\frac{\sin(k_{i}^{s2}\left(r_{i}-b\right))}{r_{i}},a<r_{e}<b,\;E_{i}>V_{i} \end{array}\right.$$

with $k_{i}^{c}=\sqrt{2m_{ic}^{*}E_{i}/\hbar^{2}}$, $k_{i}^{s1}=\sqrt{2m_{is}^{*}\left(V_{i}-E_{i}\right)/\hbar^{2}}$ and $k_{i}^{s2}=\sqrt{2m_{is}^{*}\left(E_{i}-V_{i}\right)/\hbar^{2}}$. The equivalent expressions for the type II CSQD structure differ for electrons and holes:

-electron case

(8) $$R_{n,l}^{e}\left(r_{e}\right)=\left\{\begin{array}{c} A_{e}^{c}\frac{\sinh(k_{e}^{c1}r_{e})}{r_{e}},\;0<r_{e}<a,\;E_{e}<V_{e}\\ A_{e}^{c}\frac{\sin(k_{e}^{c2}r_{e})}{r_{e}}\;,a<r_{e}<b,\;E_{e}>V_{e}\\ A_{e}^{s}\frac{\sin(k_{e}^{s}\left(r_{e}-b\right))}{r_{e}},\;\;a<r_{e}<b,\; \end{array}\right.$$

with $k_{e}^{s}=\sqrt{2m_{es}^{*}\left(E_{e}\right)/\hbar^{2}}$, $k_{e}^{c1}=\sqrt{2m_{ec}^{*}\left(V_{e}-E_{e}\right)/\hbar^{2}}$ and $k_{e}^{c2}=\sqrt{2m_{ec}^{*}\left(E_{e}-V_{e}\right)/\hbar^{2}}$.

-hole case

(9) $$R_{n,l}^{h}\left(r_{h}\right)=\left\{\begin{array}{c} A_{h}^{c}\frac{\sin(k_{h}^{c}r_{h})}{r_{h}},\;0<r_{h}<a\;\\ A_{h}^{s}\frac{\sinh(k_{h}^{s1}\left(r_{h}-b\right))}{rh}\;,\;a<r_{h}<b,\;E_{h}<V_{h}\\ A_{h}^{s}\frac{\sin(k_{h}^{s2}\left(r_{h}-b\right))}{r_{h}},\;a<r_{h}<b,\;E_{h}>V_{h} \end{array}\right.$$

with $k_{h}^{c}=\sqrt{2m_{hc}^{*}E_{h}/\hbar^{2}}$, $k_{h}^{s1}=\sqrt{2m_{hs}^{*}\left(V_{h}-E_{h}\right)/\hbar^{2}}$ and $k_{h}^{s2}=\sqrt{2m_{hs}^{*}\left(E_{h}-V_{h}\right)/\hbar^{2}}$. $A_{i}^{j}(j=core,$ shell, i=e,h) are the normalization constants determined by the condition $\langle \Psi_{i}\left(r_{i}\right)|\Psi_{i}\left(r_{i}\right)\rangle =1$.

The transcendental equation for calculating the energy of the electron and hole ground states ($E_{i}$), is given by the continuity of the radial wave function and its probability current at the core surface:

(10) $$\left\{\begin{array}{c} {\left.{\;\psi }_{i}\left(core\right)\right|}_{r_{i}=a_{e}}={\left.{\;\psi }_{i}\left(shell\right)\right|}_{r_{i}=a_{e}}\\ {\left.\frac{1}{m_{1i}^{*}}\frac{d{\;\psi }_{i}\left(core\right)}{dr_{i}}\right|}_{r_{i}=a_{e}}={\left.\frac{1}{m_{2i}^{*}}\frac{d{\;\psi }_{i}\left(shell\right)}{dr_{i}}\right|}_{r_{i}=a_{e}} \end{array}i=e,\;h\right.$$

### 2.2. Effects of Strain on Band Structures

A spherical CSQD structure will be subject to a dilatational eigenstrain β due to lattice mismatch at the core and shell interface. This strain field is given as [8,50]:

(11) $$\beta_{rr}^{c}=\beta_{\theta \theta }^{c}=\beta_{\varphi \varphi }^{c}=\beta ra\frac{2(3+3ra+ra^{2})}{3{\left(1+ra\right)}^{3}}\frac{\left(1-2pr\right)}{\left(1-pr\right)},$$

(12) $$\begin{array}{ccc} \beta_{rr}^{s} & = & \beta (2\left(1-2pr\right)-2{\left(1+ra\right)}^{3})\\ & & \frac{\left[1-2pr-3(1+pr)\ln(1+ra)\right]}{9{\left(1+ra\right)}^{3}\left(1-pr\right)\left(pr+pr^{2}+pr^{3}/3\right)}, \end{array}$$

(13) $$\begin{array}{ccc} \beta_{\theta \theta }^{s} & = & \beta_{\varphi \varphi }^{s}=\beta \left(2\left(1-2pr\right)-{\left(1+ra\right)}^{3}\right)\\ & & \frac{\left[2-4pr+3(1+pr)\ln(1+ra)\right]}{9{\left(1+ra\right)}^{3}\left(1-pr\right)\left(pr+pr^{2}+pr^{3}/3\right)}, \end{array}$$

where ra=(b−a)/a, pr is the Poisson ratio, $\beta =\left(L_{s}-L_{c}\right)/L_{c}$ denotes the lattice mismatch constant of the core ($L_{c}$) and shell ($L_{s}$). The strain modified band edges of conduction ($V_{CB}$) and valence bands ($V_{VB}$) of core and shell material are written as [57,58]:

(14) $$\begin{array}{ccc} V_{CB}^{c,s} & = & E_{CB}^{c,s}+a_{CB}^{c,s}\beta_{H}^{c,s}, \end{array}$$

(15) $$\begin{array}{ccc} V_{VB}^{c,s} & = & E_{VB}^{c,s}+a_{CB}^{c,s}\beta_{H}^{c,s}+\frac{b^{c,s}}{2}\beta_{B}^{c,s}, \end{array}$$

with

(16) $$\begin{array}{ccc} \beta_{H}^{c,s} & = & \beta_{rr}^{c,s}+\beta_{\theta \theta }^{c,s}+\beta_{\varphi \varphi }^{c,s}, \end{array}$$

(17) $$\begin{array}{ccc} \beta_{B}^{c,s} & = & -\beta_{H}^{c,s}\frac{2C_{12}^{c,s}+C_{11}^{c,s}}{C_{12}^{c,s}-C_{11}^{c,s}}, \end{array}$$

where $\beta_{B}^{c,s}$ and $\beta_{H}^{c,s}$ are biaxial and hydrostatic strain, respectively. $E_{CB}^{c,s}$ and $E_{VB}^{c,s}$ are the conduction and valence band edges energies without strain. $a_{CB}^{c,s}$, $a_{VB}^{c,s}$ and $b^{c,s}$ are the deformation potential. $C_{12}^{c,s}$ and $C_{11}^{c,s}$ are elastic constants.

Considering the above expressions, the confining potentials $V_{e}$ and $V_{h}$ in Equations (3)–(5) are:

(18) $$V_{e}=\left|V_{CB}^{c}-V_{CB}^{s}\right|,\;\;V_{h}=V_{VB}^{c}-V_{VB}^{s}.$$

The absolute value of $V_{e}$ should be used because of $V_{CB}^{c}<V_{CB}^{s}$ in the case of type I structure. For type II structure, we have $V_{CB}^{c}>V_{CB}^{s}$.

### 2.3. Donor Impurity in Core/Shell Structure

Now, let us consider an impurity confined in a spherical CSQD. The Hamiltonian of this impurity in the presence of the magnetic field, described by the Lorenz gauge relating the vector potential to this field by the well-known relation $\vec{A}=\frac{1}{2}\vec{B}\times \vec{r}$, can be expressed by:

(19) $$H_{D}=-\frac{\hbar^{2}}{2m_{e}^{*}}\Delta_{e}+M-\frac{e^{2}}{\varepsilon r_{ed}}+V_{e},$$

where $r_{ed}=\left|\vec{r_{e}}-\vec{d}\right|=\sqrt{r_{e}^{2}+d^{2}-2r_{e}d\cos\left(\theta \right)}$ is the electron impurity distance. *M* is the magnetic operator given as [59]:

(20) $$M=\frac{e^{2}B^{2}}{8\;m_{e}^{*}c}r_{e}^{2}\sin^{2}\left(\theta \right)-\frac{eB\hbar }{\;2m_{e}^{*}c}L_{ze},$$

where $L_{ze}$ denotes the *z* component of the angular momentum.

By using as a unit of length the effective Bohr radius ${\tilde{a}}_{D}=\hbar^{2}\tilde{\varepsilon }/e^{2}{\tilde{m}}_{e}^{*}$, the donor effective Rydberg ${\tilde{R}}_{D}^{*}=\hbar^{2}/2{\tilde{m}}_{e}^{*}{\tilde{a}}_{D}^{2}$ for energy, with ${\tilde{m}}_{e}^{*}=\sqrt{m_{e}^{c*}m_{e}^{s*}}$ is the electron mean effective mass. Indeed, in CSQD structures, the electron wavefunction extends across both the core and shell regions, and the electron effective mass can vary spatially due to the different material properties. Therefore, by taking the square root of the electron-effective mass in the core and shell regions, we aim to describe the mean behavior of the electron within these areas. $\tilde{\varepsilon }=\sqrt{\varepsilon^{c}\varepsilon^{s}}$ is the mean relative dielectric constant. The Hamiltonian 19 becomes:

(21) $$H_{D}=-\frac{{\tilde{m}}_{e}^{*}}{m_{e}^{i*}}\Delta_{e}+M-\frac{2\tilde{\varepsilon }}{\varepsilon^{i}r_{ed}}+V_{e};\;\;i=core,shell$$

The magnetic operator *M* can be simplified by using the usual dimensionless parameter $\gamma =\frac{\hbar \omega_{c}}{2{\tilde{R}}_{D}^{*}}$ characterizing the strength of the magnetic field ($\omega_{c}=\frac{eB}{{\tilde{m}}_{e}^{*}c}$ is the effective cyclotron frequency), therefore *M* can be written as:

(22) $$M=\frac{{\tilde{m}}_{e}^{*}}{m_{e}^{i*}}\left(-\gamma L_{ze}+\frac{1}{4}\gamma r_{e}^{2}\sin^{2}\left(\theta \right)\right);\;\;i=core,shell$$

The impurity energy $E_{D}$ and wave functions $\Psi_{D}$ can be calculated from the Schrödinger equation: $H_{D}\Psi_{D}=E_{D}\Psi_{D}.$ This equation cannot be solved analytically. Thus, the solution must be obtained numerically by using, in our case, a variational method. This method requires a good choice of wavefunction containing variational parameters. To justify the choice of the trial wave function, we performed another calculation using the finite element method (FEM) through Comsol Multiphysics software, which is a good approach to solving the governing Partial Differential Equations (PDEs) numerically [60]. The trial wave function was selected as follows [12,61]:

(23) $$\Psi_{D}=NR_{n,l}^{e}\left(r_{e}\right)e^{-\alpha r_{ed}}e^{-\eta \frac{\gamma^{2}}{4}\left(r_{e}^{2}\sin^{2}\left(\theta \right)\right)}$$

$e^{-\alpha r_{ed}}$ represents the Coulomb correlations between the electron and the donor impurity. $e^{-\eta \frac{\gamma^{2}}{4}\left(r_{e}^{2}\sin^{2}\left(\theta \right)\right)}$ describes the effect of the magnetic field. α and η are the nonlinear variational parameters to be determined to minimize the mean values of energies $E_{D}$:

(24) $$E_{D}=\min_{\alpha ,\eta }\frac{\langle \Psi_{D}|H_{D}|\Psi_{D}\rangle }{\langle \Psi_{D}|\Psi_{D}\rangle }$$

The donor binding energy ($E_{b}$) of the ground state, in the presence of the magnetic field, is defined as:

(25) $$E_{b}\left(\gamma \right)=E_{e}\left(\gamma \right)-E_{D}\left(\gamma \right)$$

The formula of the diamagnetic susceptibility $\chi_{dia}$ of the donor impurity (a key parameter characterizing the response of donor to magnetic fields) confined in a CSQD is given by [62]:

(26) $$\chi_{dia}=-\frac{{\tilde{m}}_{e}^{*}e^{2}}{6m_{e}^{i*}\varepsilon c^{2}}\langle {\left(\vec{r_{e}}-\vec{d}\right)}^{2}\rangle ;\;\;i=core,shell$$

## 3. Results and Discussion

This work aims to investigate the effects of the magnetic field and the structure of the CSQD on the binding energy and diamagnetic susceptibility of a donor impurity confined in its interior. To meet the objectives of our study, we have chosen two different core/shell QD compounds each other: *CdTe/ZnS* and *CdTe/CdS*, which are type I and type II core/shell, respectively. The physical parameters used in the current study are listed in Table 1.

**Figure 2:**
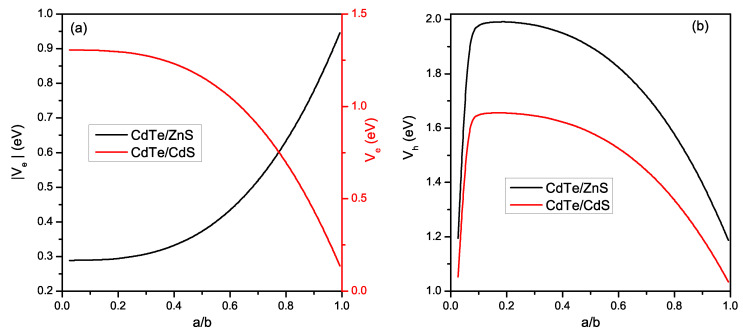
Evolution of the confinement potential of the electron (**a**) and hole (**b**) under the effect of the strain field as a function of the core/shell ratio a/b for *CdTe/ZnS* (type I) and *CdTe/CdS* (type II).

First, it is imperative to note that for the core/shell QD, the variation of the electronic and optical properties of these nanostructures is strongly influenced by the effect of the strain field on the band structure (often overlooked in several theoretical studies). For this reason, we start our study by investigating the strain effects on the confinement potential of the electron ($V_{e}$) and hole ($V_{h}$) for *CdTe/ZnS* and *CdTe/CdS* CSQD materials. In Figure 2, we present the variation of these confinement potentials as a function of the ratio a/b. For the *CdTe/ZnS* material (type I), we use the absolute value of $V_{e}$ because $V_{CB}^{c}<V_{CB}^{s}$. As a first remark, we notice that the strain has a significant impact on both confinement potentials ($V_{e}$ and $V_{h}$). Regarding $V_{e}$ (Figure 2a), we can notice that the electron confinement potential of *CdTe/ZnS* monotonically augments with the increase of the ratio a/b. In contrast, the $V_{e}$ of *CdTe/CdS* reduces as a/b increases. Concerning $V_{h}$ (Figure 2b), we have found that both *CdTe/ZnS* and *CdTe/CdS* hole confinement potential follow the same evolution (though their values differ), where both of them quickly increase until reaching a maximum at a/b = 0.14 for *CdTe/ZnS* and at a/b = 0.09 for *CdTe/CdS*; after which they decrease.

In addition to the strain effects, the optoelectronic properties of these nanostructures depend on the locations of the charge carriers, which are related to the nature of the material and their appropriate band offsets. In fact, according to the wave functions established in different regions (related to $E_{i}<$ or $>V_{i}$) (Equations (7)–(9)), the charge carriers can leave the core to shell materials, or vice versa, when the core radius reaches the critical values $a_{c}^{i}$ for a given shell size *b*. These values can be obtained numerically when the energy of the charge carriers reaches the band offsets ($E_{i}=V_{i}$). This very interesting point was very carefully examined in our previous work [56]. In Table 2, we give the critical radii of the electron and hole corresponding to the two shell sizes used in our investigation for both systems (*CdTe/ZnS* and *CdTe/CdS*). The difference in the $a_{c}^{i}$ values between the two structures is attributed to the physical parameters, the band alignment of the materials, and the potential confinements under strain.

**Figure 3:**
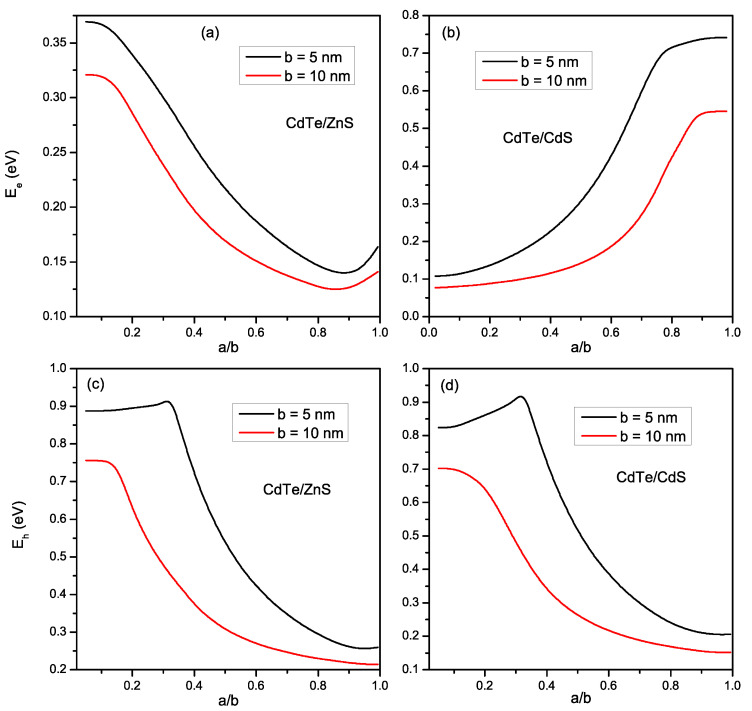
Variation of the electron energy (**a**,**b**) and the hole energy (**c**,**d**) for *CdTe/ZnS* and *CdTe/CdS* respectively, as a function of a/b ratio, for *b* = 5, and 10 nm.

In Figure 3a,b, we plot the electron energy confined in a *CdTe/ZnS* and *CdTe/CdS* spherical CSQD, respectively, as a function of the ratio a/b for two shell radius values *b* = 5 and 10 nm. For *CdTe/ZnS* (type I), we found that the electron energy decreases with increasing shell radius *b* because of the confinement effect and also of the strain field impact on the electron confinement potential as shown in Figure 2a. For a fixed outer radius, it can also be seen that $E_{e}$ rises when the a/b ratio decreases because the confinement weakens (strengthens) as core size increases (reduces), and therefore this change in $E_{e}$ appears. The electron energy comprises two regions separated by the critical radius ($a_{c}^{e}$) (depending on the shell radius (Table 2)): one is the bound state ($E_{i}<V_{i}$); and the other is the unbound state ($E_{i}>V_{i}$), where the energy tends towards a constant value as long as the radius of the core exceeds the critical radius. Concerning *CdTe/CdS* (type II) (Figure 3b), it is remarkable that the behavior of the electron energy is opposite to that found for *CdTe/ZnS*, due to the band alignment difference between the two structures (Figure 1a,b, and Equations (3) and (4)), where the electron is initially trapped on the shell in this case. The variations of the hole energies for the *CdTe/ZnS* and *CdTe/CdS* materials are plotted in Figure 3c,d, respectively. We can see that the two energies behave in the same way ($E_{h}$ decrease as *b* increases) due to the same band alignment related to the hole position for both structures (Equations (3) and (5)). For a given shell radius, we remark that, for the bound state ($E_{h}<V_{h}$), the hole energies reduce when a/b augments. Regarding the unbound state ($E_{h}>V_{h}$), we find that, $E_{h}$ is slightly decreased when *a* becomes inferior to $a_{c}^{h}$. This evolution is attributed to the variation in the hole confinement potential under the strain effect (Figure 2b).

**Figure 4:**
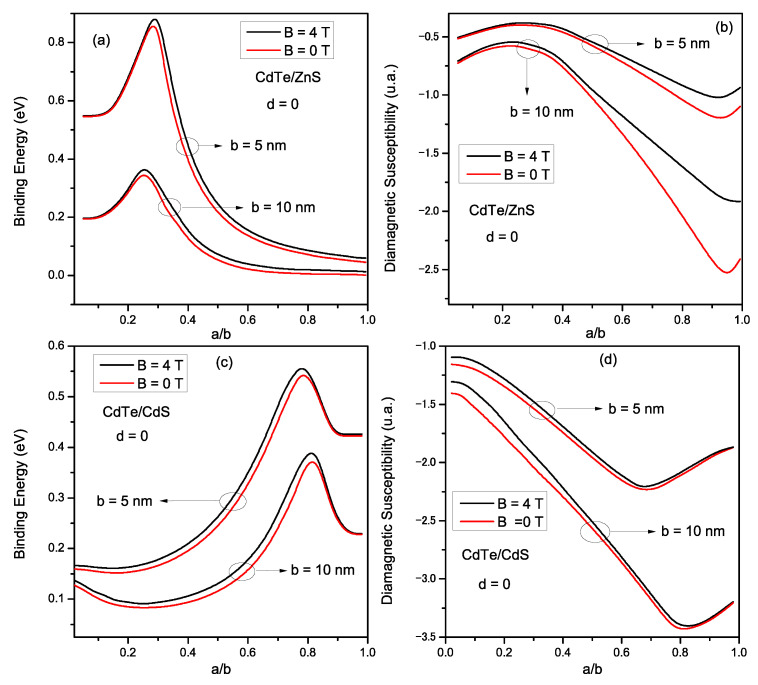
Variation of the binding energy (**a**,**c**) and diamagnetic susceptibility (**b**,**d**) for *CdTe/ZnS* and *CdTe/CdS* respectively, as a function of a/b ratio; for *B* = 0 and 4 T, and for *b* = 5 and 10 nm.

**Figure 5:**
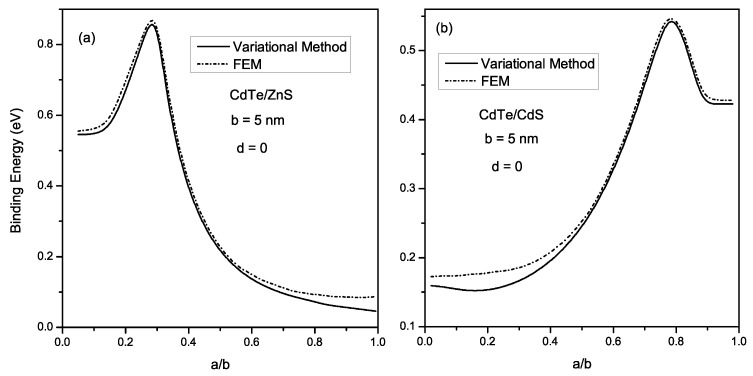
Variation of the binding energy for *CdTe/ZnS* (**a**) and *CdTe/CdS* (**b**), calculating via variational approach (solid line) and via FEM implemented in COMSOL Multiphysics (dashed line); as a function of a/b ratio for *b* = 5 nm.

To look further, we consider now, a donor impurity confined within the CSQD. In Figure 4, we present the dependence of the binding energy ($E_{b}$) and the diamagnetic susceptibility ($\chi_{dia}$) of the impurity as a function of the a/b ratio. Calculations were performed for two CSQD sizes: *b* = 5 nm and 10 nm. In addition, for each size, the system was considered without a magnetic field (red curves) and immersed in a magnetic field (black curves). Figure 4a shows that $E_{b}$ increases as the core grows from small sizes until it reaches a maximum value, corresponding to ${\left(a/b\right)}_{cri}$. This values are ${\left(a/b\right)}_{cri}$ = 0.26 and ${\left(a/b\right)}_{cri}$ = 0.3 for *b* = 5 nm and *b* = 10 nm respectively. For larger cores, $E_{b}$ decreases monotonically. This well-known turnover behavior is related to the change in the geometrical confinement of the CSQD; as the core radius decreases, the Coulomb interaction between the electron and the impurity becomes more important, and thus $E_{b}$ increases. On the other hand, when the core radius drops below $a_{c}^{e}$, the electron is forced to leave the core to the shell, i.e., the electronic wave function is extended to the shell and therefore, the impurity $E_{b}$ decreases. Figure 4c shows the variation of the impurity $E_{b}$ for *CdTe/CdS* (type II) is displayed as a function of the a/b ratio, under the same conditions used in Figure 4a. It is remarkable that for a given shell radius, $E_{b}$ increases with the core radius augmentation and shows a maximum at ${\left(a/b\right)}_{cri}$ = 0.77 for *b* = 5 nm and at ${\left(a/b\right)}_{cri}$ = 0.80 for *b* = 10 nm. This behavior can be explained by the strength of electronic wave function localization inside the shell when the core size augments, which enhances the electron-impurity interaction. Therefore, $E_{b}$ rises. When *a* reaches the $a_{c}^{e}$, the electron leaves the shell to the core, which leads to a reduction in the $E_{b}$. Concerning the magnetic field (*B*) effect, applying *B* leads to an augmentation in the impurity $E_{b}$ for both structures because the magnetic field strengthens the confinement. This $E_{b}$ growth is more evident for the weak confinement (a/b tends to 1 for type I and a/b tends to 0 for type II) due to the spatial expansion of the wave function, and then the system becomes more susceptible to the *B* effect in this case. Figure 4b shows the diamagnetic susceptibility ($\chi_{dia}$) of the impurity donor within *CdTe/ZnS* material. As it is seen, $\chi_{dia}$ decreases when the shell radius increases for both CSQD sizes and magnetic field magnitudes. We also observed that when the core grows from small size, $\chi_{dia}$ slightly grows until reaching a maximal value at $a=a_{c}^{e}$, then decreases and rises once more (when *a* tends to *b*) as the core radius increases. The physical reasons for this evolution are related to the penetration of the wave function into the shell region when the core size reduces and to the variation in the electron confinement potential under the strain effect. Regarding the impurity $\chi_{dia}$ for type II structure (Figure 4d), we remark that $\chi_{dia}$ decreases to a minimum at $a=a_{c}^{e}$ and then augments when the electron moves to the core region ($E_{e}>V_{e}$). Moreover, one may observe that both structures experience an increase in $\chi_{dia}$ when *B* is applied. This increase is less important for strong confinement. In contrast, for weak confinement, the *B* effect becomes more obvious, especially in the type I case, where the field acts as an additional geometric confinement, leading to this growth found in $\chi_{dia}$ evolution. To check the validity of the choice of the trial wave function given in Equation (23), we plot in Figure 5, the evolution of the $E_{b}$ of *CdTe/ZnS* (Figure 5a) and *CdTe/CdS* (Figure 5b), as a function of the radii ratio a/b, and for *b* = 5 nm; by using the variational approach (solid line) and FEM (dashed line). As can be remarked from this figure, the results of both methods are close with a small difference when *a* tends to *b* for *CdTe/ZnS* and *a* tends to 0 for *CdTe/CdS*. These obtained results confirm the appropriate choice of the trial wave-function describing the interaction between the electron and impurity inside the CSQD.

**Figure 6:**
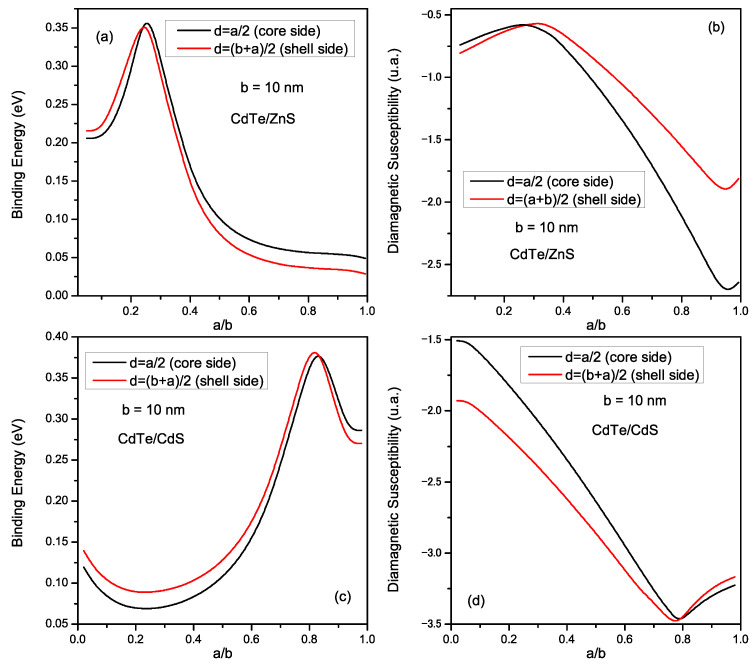
(**a**,**c**) Variation of the binding energy; (**b**,**d**) variation of the diamagnetic susceptibility for *CdTe/ZnS* and *CdTe/CdS* materials, as a function of a/b ratio; for *b* = 10 nm, and for two impurity positions (core or shell sides).

In Figure 6, we present the dependence of the binding energy ($E_{b}$) and the diamagnetic susceptibility ($\chi_{dia}$) of the impurity as a function of the a/b ratio, for *b* = 10 nm. The calculations were performed for *CdTe/ZnS* (upper figures) and the *CdTe/CdS* (lower figures). In addition, the curves were performed for two impurity positions: on the core side d=a/2 (black curves) and the shell side d=(b+a)/2 (red curves). For the case of *CdTe/ZnS*, remarkably, the impurity position strongly affects $E_{b}$. For $E_{e}<V_{e}$ where the electron is in the core, we notice that $E_{b}$ is more pronounced when the impurity is placed on the core side (d=a/2), and this is normal because the donor is close to the electron which leads to reinforcement in the Coulomb interaction. Consequently, the binding energy becomes more significant. For $E_{e}>V_{e}$, where the electron leaves the core to the shell, the situation changes, and $E_{b}$ becomes more significant when the impurity is localized in the shell (d=(b+a)/2), i.e., the electron joins the donor in the shell and therefore $E_{b}$ increases. For the case of *CdTe/CdS* (Figure 6c), we find an inverse behavior of $E_{b}$ compared to that found for *CdTe/ZnS*, due to the type II structure where the electron is normally found in the shell, thus $E_{b}$ is more pronounced when the impurity is placed in the shell. In contrast, when the donor is placed in the core, $E_{b}$ becomes more important when the electron moves to the core ($E_{e}>V_{e}$). In conclusion, $E_{b}$ is more important for both cases when the electron and the impurity are trapped in the same region. Always with the impurity positions effect, we plot in Figure 6b,d the variation of the donor diamagnetic susceptibility for *CdTe/ZnS* and (*CdTe/CdS*) respectively. These figures show that $\chi_{dia}$ is more pronounced when the impurity and the electron are confined in different regions (electron in the shell and donor in the core or vice versa).

**Figure 7:**
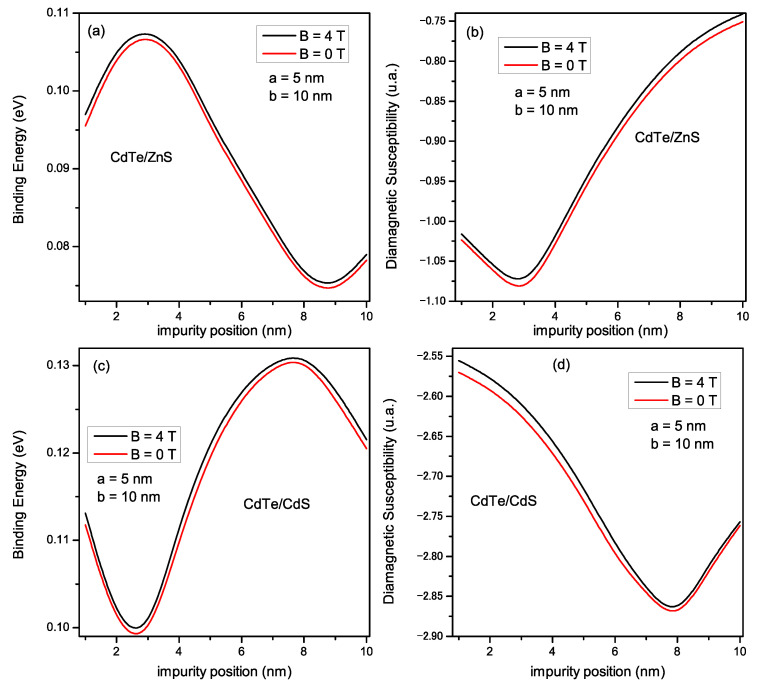
Variation of the binding energy (**a**,**c**) and diamagnetic susceptibility (**b**,**d**) for *CdTe/ZnS* and *CdTe/CdS* respectively, as a function of impurity positions; for *a* = 5 nm and *b* = 10 nm, and for *B* = 0 and 4 T.

In Figure 7, we present the dependence of the binding energy ($E_{b}$) and diamagnetic susceptibility ($\chi_{dia}$) of the impurity as a function of the donor position, for the type I structure (figures above) and for the type II structure (figures below). The core radius *a* = 5 nm and the shell radius *b* = 10 nm were considered for both structures. The position of the donor impurity sweeps the structure from 0 to *b*. Moreover, the curves were performed for two magnetic field values: *B* = 0 T (red curves) and *B* = 4 T (black curves). We observe that, for the type I structure, $E_{b}$ increases to a maximum when the impurity is placed at d=a/2, then decreases to a minimum value at d=(a+b)/2 (shell center), and increases slightly when the donor approaches the shell edge. As for type II, it is observed that $E_{b}$ decreases to a minimum at d=a/2 (the impurity position is close to the core center), then increases to a maximum at d=(a+b)/2, and then decreases as the donor approaches the shell edge. Regarding the variation of the diamagnetic susceptibility of the donor (Figure 7b,d), our results indicate that for type I (type II), $\chi_{dia}$ obtains a minimum (maximum) at d=a/2 (d=(a+b)/2) and then increases (decreases) when the impurity approaches the shell edge (core center). We can also observe that applying the magnetic field increases $E_{b}$ and $\chi_{dia}$ for both structures.

## 4. Conclusions

We have theoretically investigated the electronic properties of a donor impurity confined in a *CdTe/ZnS* (Type I) and *CdTe/CdS* (Type II). We take into account the effects: (i) the strain field caused by lattice mismatch, (ii) the size of the structure (altering the core and shell radii), (iii) the magnetic field, (iv) the location of the impurity (from the core center to the shell edge). Our results show that the strain field significantly impacts the structure bands (conduction and valence bands) and, thus, the electron and hole energy spectrum. We have also shown that the impurity’s diamagnetic susceptibility and binding energy for the type I structure have inverse behaviors compared to those for the type II structure—furthermore, the impurity’s diamagnetic susceptibility and binding energy increase when the magnetic field is applied. Our present investigation can be considered as a contribution to studying and understanding the characteristics of this class of heterostructures, especially the impact of the strain field on the structure bands, and critical radii that allow particles to pass from one region (core or shell) to another. Indeed, because of the nature of the behaviors of these two structures (one is opposite to the other), these materials can be combined in order to wider their optical and electronic properties and therefore offer more optoelectronic features that can be exploited for many fields of applications.

## Figures and Tables

**Table 1:** Physical parameters of the studied materials [63,64,65].

	*CdTe*	*CdS*	*ZnS*
$m_{e}^{*}/m_{0}$	0.09	0.14	0.22
$m_{h}^{*}/m_{0}$	0.63	0.68	1.42
ε	10.2	9.8	8.3
*L* (nm)	0.6481	0.5825	0.5410
$C_{11}$ ($10^{11}$dyn/cm)	5.35	7.7	10.2
$C_{12}$ ($10^{11}$dyn/cm)	3.69	5.39	6.46
$E_{CB}$ (eV)	−3.6	−3.69	−2.64
$E_{VB}$ (eV)	−5.1	−6.12	−6.27
$a_{CB}$ (eV)	−3.96	-27.1	−4.49
$a_{VB}$ (eV)	0.55	0.92	1.83
*b* (eV)	−1.1	−1.18	−1.39
pr	0.4		

**Table 2:** Critical values of core size at which electron or hole move to the other region, for different shell sizes, and for both structures.

	*CdTe/ZnS*		*CdTe/CdS*	
	$a_{c}^{e}$ **(nm)**	$a_{c}^{h}$ **(nm)**	$a_{c}^{e}$ **(nm)**	$a_{c}^{h}$ **(nm)**
*b* = 5 nm	1.464	0.8353	3.9337	1.1962
*b* = 10 nm	1.3737	0.8007	8.7621	1.1255

## Data Availability

Not applicable.
